# News and Notes

**Published:** 1908-04

**Authors:** 


### NEWS AND NOTES

---

The Chicago Policlinic announces a special practical course in surgery, gynecology, skin, venereal and rectal diseases and diseases of the stomach, beginning April 6, 1908, and continuing three weeks.

---

A concert is given at the Berlin Charite every Saturday between 5 and 6 p. m. for the benefit of the patients, a woman's club providing skilled musicians for the purpose.

---

The famous collection of more 2,000 dermatologic casts, owned by the late Professor Lassar, has been presented by his widow to the City of Hamburg, his native town.

---

The dedication exercises of the New Tabernacle Infirmary were held March 11, about 2,000 people being present. This building is a three-story, commodious one, which will be equipped with all modern hospital appointments, and with the increased staff, will afford distinct improvement in the hospital facilities of Atlanta.

Messrs. Lea Bros. & Co., announce the dissolution of their firm on Dec. 31st, and the continuance of the business by their successors under the title of Lea & Febiger.

---

The Alaska-Yukon-Pacific Exposition will be held at Seattle, Wash., opening June 1, and closing October 15, 1909.

---

Lieutenant Colonel Blair D. Taylor, recently of the General Hospital at Hot Springs, Ark., has been ordered to Atlanta to succeed Colonel Gray, as Chief Surgeon of the Department of the Gulf.

---

The tuberculosis exhibit of the Indiana State Board of Health has been loaned to the Richmond Health Department, and has been on exhibition in the city hall, where it has attracted much attention and favorable comment.

---

At the annual meeting of the Medical Board of Macon Hospital Association, January 29, Drs. Max Jackson, William J. Little, James H. Shorter, Henry P. Derry, Jesse E. Wright, Maury M. Stapler, Charles C. Herrold, John A. Selden, Samuel B. Palmer, H. H. Johnston, Johnson M. Moore, Herbert Respess and Fred L. Webb were added to the staff of the hospital.

---

### NOTICE OF EXAMINATION FOR JUNIOR INTERN TABERNACLE INFIRMARY.

Examination will be held in room 726, Candler Building, Tuesday, 8 p. m., April 21, 1908.

Term of service, eighteen (18) months, commencing May 1st.

The new Infirmary Building will soon be completed, which will give Interns excellent opportunities for practical experience.

For further information, address,

INTERN COMMITTEE,
725-6 Candler Bldg., Atlanta, Ga.

The Banks County Medical Society met March 17. Officers elected: Dr. Vinscent D. Lockhart, of Maysville, President; Dr. J. D. Rice, of Homer, Secretary and Treasurer; Dr. T. G. Underwood, of Maysville, Delegate to State Association; Dr. O. N. Harden was elected as a member. A meeting was called for May 15th.

---

### FULTON COUNTY MEDICAL SOCIETY, MARCH 5, 1908.

---

### TROPICAL ULCER.

---

Dr. Wilmerding exhibited a case of tropical ulcer, the history of which we will report in full in our next issue.

Dr. Andrews stated that the condition is rare, and due to a virulent and contagious organism. The course is always chronic.

Dr. Wilmerding added that it appears only in the skin, the mucus membrane being free.

Dr. Hoke exhibited a case of severely crushed ankle, upon which he had operated. The injury had been so great as to cause amputation of the thigh on one side. On the other the ankle had been broken and twisted out of all normal shape, and the heel destroyed. Plastic bone operations were indicated by the skiagraphs, and these were performed, giving a serviceable foot in proper position with ankle motion in all directions. The case shows the practicability of plastic operations upon bones, even in so complicated an inter-relation as those of the ankle joint.

The following delegates to the State Association, which will meet April 15, 16, 17, in Fitzgerald, are: Drs. J. C. Olmstead, A. W. Stirling and Michael Hoke.

### THE PROGRAM FOR APRIL.

April 2. (1) "The Opsonic Contents of the Body Fluids." J. E. Paullin.

Discussion: Claude Smith, H. F. Harris.

(2) "A Demonstration of the Anatomy of the Peritoneum and the Gastro-Intestinal Tract." W. B. Armstrong.

Discussion: T. V. Hubbard, F. W. McRae, W. P. Nicholson, F. Hodgson.

April 9. (1) "A Report on One Hundred and Twenty-Two Operations on the Knee Joint." M. Hoke, C. R. Andrews.

Discussion: W. S. Goldsmith, J. L. Campbell, S. T. Barnett, F. K. Boland.

(2) "Chronic Affections of the Heart Muscle." G. P. Huguley.

Discussion: W. F. Wilmerding, R. T. Dorsey, J. H. Hines.

(3) "Nervous Diseases Caused by Alcohol and Metalic Poisons." J. Cheston King.

Discussion: J. S. Todd, M. Mc H. Hull, J. C. Olmstead, W. E. Taylor.

Lectures for the People: Drs. M. Hoke, Chairman; Ellis, H. F. Harris, Kime, Paullin, C. A. Smith, Wolff.

Minimum Fee Scale: Drs. E. C. Davis, Chairman; W. B. Armstrong, Barnett, Hoke, Hodgson, Hull, McRae, Nicholson, Roy, Taylor, Westmoreland.

Concerning a Collector: Drs. Ballenger, Chairman; Strickler, Huguely, Sthephens, Hutchins.

Committee on Membership: Drs. LeConte, Chairman; Wilmerding, White.

---

The following committees have been appointed by Dr. A. W. Stirling for the year 1908:

Board of Censors: Drs. E. C. Davis (1 yr. Ch.), J. C. Olmstead (2 yrs.), W. B. Emery (3 yrs.).

Publication Committee: E. G. Ballenger, Chairman; E. Bates Block; W. S. Goldsmith.

Public Health and Legislation: Drs. C. W. Strickler, Chairman; C. A. Smith, L. B. Clarke.

Case Committee: Drs. W. B. Armstrong, Chairman; E. G. Ballenger, R. L. Dorsey, J. H. Hines, C. D. Roy, J. L. Campbell, W. S. Goldsmith, R. B. Ridley, Jr., A. Smith.

Programme Committee: Drs. A. W. Stirling, Chairman; J. W. Duncan, J. Ross Simpson.

---

The Medical Board of Grady Hospital has decided that in the future the medical and surgical services of the hospital should be divided and separate.

Before this time the services have been combined, the same house physicians serving in both medical and surgical capacities.

There have been five house physicians elected by competitive examination from young medical graduates, the term of service of each being two years. According to the new plan this number will be increased to eight, of which three will be chosen for exclusive medical service and five solely for surgical work of the institution. The ambulence men will be surgeons.

---

Dr. J. M. Sadler, a prominent Physician of Montgomery, Ala., died March 7, while attending the Montgomery Medical Society. He had just entered the hall, where he spoke to his friends and sat down. In a few moments he died of hemorrhage of the brain. Dr. Sadler was 50 years of age, and was a native of South Carolina.

---

Dr. Dunbar Roy has examined 900 employees of the Southern Railway, testing their vision for color blindness, and their hearing abilities. This work has been done under the direction of Dr. Applegate, chief surgeon of the railway, for the purpose of lessening wrecks, many of which are attributed to failure of employees to see signals aright. Dr. Roy has found very few who fail to come up to the standard required by the road.

---

### INTERNATIONAL CONGRESS OF TUBERCULOSIS.

---

The following prizes are offered by the International Congress of Tuberculosis, which meets in Washington, D. C., next September:

A prize of $1,000 is offered for the best evidence of effective work in the prevention or relief of tuberculosis by any voluntary association since the last international congress in 1905. In addition to the prize of $1,000, two gold medals and three silver medals will be accompanied by diplomas or certificates of award.

2. A prize of $1,000 is offered for the best exhibit of an existing sanatorium for the treatment of curable cases of tuberculosis among the working classes. In addition to the prize of $1,000, two gold medals and three silver medals will be awarded.

3. A prize of $1,000 is offered for the best exhibit of a furnished house for a family or group of families of the working class, designed in the interest of the crusade against tuberculosis. In addition to the prize of $1,000, two gold medals and three silver medals will be awarded.

4. A prize of $1,000 is offered for the best exhibit of a dispensary or kindred institution for the treatment of the tuberculous poor. In addition to the prize of $1,000, two gold medals and three silver medals will be awarded.

5. A prize of $1,000 is offered for the best exhibit of a hospital for the treatment of advanced pulmonary tuberculosis. In addition to the prize of $1,000, two gold medals and three silver medals will be awarded.

6. The Hodgkins fund prize of $1,500 is offered by the Smithsonian Institution for the best treatise that may be submitted on "The Relation of Atmospheric Air to Tuberculosis."

7. Prizes for educational leaflets:

A prize of $1,000 is offered for the best educational leaflet submitted in each of the seven classes defined below. In addition to the prize of $1,000, a gold medal and two silver medals will be awarded in each class.

Competitors must be entered under assumed names.

A. For adults generally (not to exceed 1,000 words).

B. For teachers (not to exceed 2,000 words).

C. For mothers (not to exceed 1,000 words).

D. For indoor workers (not to exceed 1,000 words).

E. For dairy farmers (not to exceed 1,000 words).

F. For school children in grammar school grades (not to exceed 500 words).

G. Pictorial booklet for school children in primary grades and for the nursery.

8. A gold medal and two silver medals are offered for the best exhibits sent in by any States of the United States, illustrating effective organization for the restriction of tuberculosis.

9. A gold medal and two silver medals are offered for the best exhibits sent by any state or country (the United States excluded), illustrating effective organization for the restriction of tuberculosis.

10. A gold medal and two silver medals are offered for each of the following exhibits:

A. For the best contribution to the pathological exhibit.

B. For the best exhibit of laws and ordinances in force June 1, 1908, for the prevention of tuberculosis by any state of the United States. Brief required.

C. For the best exhibit of laws and ordinances in torce June 1, 1908, for the prevention of tuberculosis by any state or country (the United States excluded). Brief required.

D. For the best exhibit of laws and ordinances in force June 1, 1908, for the prevention of tuberculosis by any municipality in the world. Brief required.

E. For the society engaged in the crusade against tuberculosis having the largest membership in relation to population. Brief required.

F. For the plans which have been proven best for raising money for the crusade against tuberculosis. Brief required.

G. For the best exhibit of a passenger railway car in the interest of the crusade against tuberculosis. Brief required.

H. For the best plans for employment for arrested cases of tuberculosis. Brief required.

11. Prizes of two gold medals and three silver medals will be awarded for the best exhibit of a workshop or factory in the interest of the crusade against tuberculosis.

---

### PRELIMINARY PROGRAM OF THE ELEVENTH ANNUAL MEETING OF THE AMERICAN GASTRO-ENTEROLOGICAL ASSOCIATION TO BE HELD AT CHICAGO, ILL., JUNE 1 AND 2, 1908.

---

1. President's address. J. P. Sawyer, Cleveland.

2. A new method of ascertaining the permeability of the pylorus. Max Einhorn, New York.

3. Ischochymia. F. H. Murdoch, Pittsburgh.

4. An explanation of the motor activities of the alimentary canal in terms of the myenteric Reflex. Walter B. Cannon, Boston.

5. (a) Further observations on the chemic co-ordination existing between the salivary glands and the secretion of the stomach.

(b.) Effect of splenectomy on the gastric secretion. J. C. Hemmeter, Baltimore.

6. Cholecystitis. H. W. Bettmann, Cincinnati.

7. Notes of progress in gastroenterology. A. L. Benedict, Buffalo.

8. The nervous influence on the production of sugar in the body. J. J. R. MacLeod, Cleveland.

9. The behavior of some indigestible carbohydrates in the alimentary tract. Lafayette B. Mendel, New Haven.

10. A comparison of the guic and benzidin tests for invisible hemorrhage in diseases of the digestive organs. Franklin W. White, Boston.

11. Paper. J. Kaufmann, New York.

12. Intestinal sand—one of its sources. Jesse S. Myer, Jerome E. Cook, St. Louis.

13. Some recent experiences with gastric ulcer. Wm. Gerry Morgan, Washington.

14. Pathology of malignant growths. W. T. Howard, Cleveland.

15. Gastromyxorrhea. J. Friedenwald, Baltimore.

---

The Seventeenth Annual Meeting of the Association of Military Surgeons of the United States will be held in Atlanta October 6-9, 1908. The officers of this Association, which will be heartily welcomed to Atlanta, are: Asst. Surg., Gen. George Tully Vaughan, P. H. and M. H. S., Washington, D. C., President; Rear Ad. Presley M. Rixey, U. S. N., Washington, D. C., First Vice-President; Col. Jas. H. Weaver, N. G. Pa., Norristown, Pa., Second Vice-President; Col. Wm. C. Gorgas, U. S. M., Ancon, Canal Zone, Panama, Third Vice-President; Major James E. Pilcher, U. S. V., Carlisle, Pa., Secretary; Major Herbert A. Arnold, N. G. Pa., Ardmore, Pa., Treasurer; Capt. J. C. DeVries, N. G., N. Y., Wakins Glen, N. Y., Asst. Secretary.

Richard Douglas, M. D., one of the South's leading surgeons, died of Bright's disease February 19th, at his home, in Nashville, Tenn. He was professor of gynecology and secretary of the faculty of the Medical Department of Vanderbilt University. Dr. Douglas was a man of much force and determination, an able teacher, a skillful surgeon with an enviable reputation and many enthusiastic admirers.

---

### HIS EYES OPENED.

"Why is she getting a divorce?"

"On the grounds of misrepresentation. She says that before they were married he claimed to be well off!"

"And what does he say?"

"He says he was, but didn't know it."

